# Use of mobile phone consultations during home visits by Community Health Workers for maternal and newborn care: community experiences from Masindi and Kiryandongo districts, Uganda

**DOI:** 10.1186/s12889-015-1939-3

**Published:** 2015-06-18

**Authors:** Richard Mangwi Ayiasi, Lynn Muhimbuura Atuyambe, Juliet Kiguli, Christopher Garimoi Orach, Patrick Kolsteren, Bart Criel

**Affiliations:** Department of Community Health and Behavioural Sciences, Makerere University College of Health Sciences, School of Public Health, P.O Box 7072, Kampala, Uganda; Institute of Tropical Medicine, Nationalestraat 155, B 2000 Antwerp, Belgium

**Keywords:** Village health teams, Home visits, Mobile phone consultation, Maternal and newborn care practices

## Abstract

**Background:**

Home visits by Community Health Workers [In Uganda Community Health Workers are given the collective term of Village Health Teams (VHTs). Hereafter referred to as VHTs] is recommended to improve maternal and newborn care. We investigated perceived maternal and newborn benefits of home visits made by VHTs, combined with mobile phone consultations with professional health workers for advice.

**Methods:**

A qualitative study was conducted in Masindi and Kiryandongo districts, Uganda, in December-2013 to March-2014. Study participants were drawn from the intervention arm of a randomised community-intervention trial. In-depth interviews were conducted with 20 prenatal and 16 postnatal women who were visited by VHTs; 5 group discussions and 16 key informant interviews were held with VHTs and 10 Key Informant Interviews with professional health workers. Data were analysed using latent content analysis techniques.

**Results:**

Majority women and VHTs contend that the intervention improved access to maternal and newborn information; reduced costs of accessing care and facilitated referral. Women, VHTs and professional health workers acknowledged that the intervention induced attitudinal change among women and VHTs towards adapting recommended maternal and newborn care practices. Mobile phone consultations between VHTs and professional health workers were considered to reinforce VHT knowledge on maternal newborn care and boosted the social status of VHTs in community. A minority of VHTs perceived the implementation of recommended maternal and newborn care practices as difficult. Some professional health workers did not approve of the transfer of promotional maternal and newborn responsibility to VHTs. For a range of reasons, a number of professional health workers were not always available on phone or at the health centre to address VHT concerns.

**Conclusions:**

Results suggest that home visits made by VHTs for maternal and newborn care are reasonably well accepted. Our study highlights potential benefits of combining home visits with phone consultations between VHTs and professional health workers. However, the challenge of attitudinal change among VHTs towards certain strongly culturally-embedded behavioural post-partum practices, resistance from part of the professional health workforce to collaborate with VHTs and the problematic availability of professional health workers are important systemic problems that need to be addressed.

**Trial registration:**

Current Controlled Trials NCT02084680.Registered 14 March 2014.

**Electronic supplementary material:**

The online version of this article (doi:10.1186/s12889-015-1939-3) contains supplementary material, which is available to authorized users.

## Background

Infant mortality is decreasing but neonatal mortality has remained resistant to change in low and middle income countries [1, 2]. It is evident that MDG targets for neonatal mortality in the sub Saharan region will not be achieved by the end of 2015 [3].

Effective interventions such as clean delivery, hygienic cord care, initiation of breastfeeding within the first hour of life without giving pre-lacteal feeds and maintaining the warm chain can avert up to 60 % of neonatal mortality [4, 5]. Preliminary studies conducted in Masindi and Kiryandongo districts, Uganda have shown that in the primary health care setting, limited prenatal and newborn care information is offered to women during pregnancy and the immediate newborn period [6]. Consequently, postnatal women in Masindi and Kiryandongo use potentially infectious materials for cutting the cord and bath their newborns soon after birth exposing newborns to infections and hypothermia [7]. Studies conducted elsewhere in Uganda have demonstrated similar results [8]. Non-adherence to maternal newborn care practices, in part, could be a result of limited interaction between pregnant women and professional health care workers. Home visits by Community Health Workers are suggested to mitigate this problem.

Evidence from south Asian countries like Bangladesh, India and Pakistan [9, 10] show that home visits made by Community Health Workers (CHWs) improved newborn care practices especially where access to health care is limited, [11]. In sub Saharan Africa, evidence is limited. In Ghana for example, home visits to promote recommended maternal and newborn care have shown promising results with initiation of breastfeeding within one hour and delayed bathing after six hours [12]. In a multi-country study involving Burkina Faso, South Africa and Uganda antenatal peer counselling by CHWs improved prevalence of exclusive breastfeeding [13]. In western Uganda, home visits by CHWs targeting children less than five years reportedly reduced prevalence of diarrhoeal diseases and fever as well as improved use of child health services [14].

However, engagement of CHWs to deliver effective health care is sometimes jeopardised because CHWs are usually low skilled [15] and operate far from formal health care systems [16]. Nevertheless, the rapidly expanding mobile phone technology in sub Saharan Africa provides an opportunity to bridge this gap. For example, in Zanzibar Tanzania, mobile phone text-messages and voucher components provided to pregnant women was associated with increased antenatal care attendance [17]. Elsewhere, the use of mobile phones among health staff and CHWs was widely accepted in Ethiopia [18], mobile phones improved adherence to treatment guidelines in Colombia [19] and in Indonesia, mobile phones increased access to peer information leading to higher self-efficacy among health staff [20].

In the Ugandan context CHWs are given the collective name of Village Health Teams (VHTs). In the context of this article we will refer to CHWs as VHTs. The national health policy in Uganda recognizes VHTs as constituent part of the health care system. VHTs in Uganda are assigned various tasks ranging from health education to dispensing of allopathic medicines [21]. This study aimed at exploring perceived maternal and newborn benefits of VHTs making home visits to prenatal and postnatal women and using mobile phone consultations to link VHTs to professional health care workers for further advice. Perceived benefits were explored from the perspectives of women, VHTs and health workers. Results from this study will help to interpret quantitative outcomes of the randomised community intervention trial. Quantitative results will be reported separately.

## Methods

### Study design

This was a qualitative enquiry that employed In-Depth Interviews, Key informant interviews and Group discussions. Study participants were drawn from prenatal-, postnatal women, VHT members and professional health workers. All study participants were part of the intervention arm of the community intervention study. Briefly, the intervention was a community intervention trial in which 16 health centres were randomly and equally allocated to control and intervention arms. The control arm received health educational messages routinely offered at the health facility, while the intervention arm received VHTs who conducted home visits and were equipped with mobile phones for consultation with professional health workers for advice. The community intervention trial study is registered with ClinicalTrials.gov (NCT02084680).

### Study area and population

This study was conducted in Masindi and Kiryandongo districts Uganda, from December 2013 to March 2014. This region is located 214 kilometres northwest of the capital Kampala. Eighty per cent of the population lives within five-kilometres walking distance to the nearest health centre. About 97 % of pregnant women make at least one antenatal care consultation and 52 % make four antenatal consultations, but less than 50 % deliver with the help of a skilled attendant [7]. In this region, VHTs have been selected and trained by their respective health departments on basic health promotion and community mobilization. In Uganda, community members nominate male or female volunteers to serve as VHTs. For this study VHTs were selected from pre-existing VHT members.

### Selection of study participants

Prenatal women for in-depth interviews were drawn from five of eight intervention health centres. The number of five health centres was considered logistically feasible. Twenty prenatal women (four from each health centre) who were antenatal attendees and enrolled into the intervention arm were purposively selected to participate in the in-depth interviews. Purposive selection of prenatal women was preferred to ensure synchronised postnatal in-depth interviews with the same women. Therefore, inclusion into the first in-depth interview was based on prenatal women having similar expected dates of delivery. Records of expected dates of delivery were obtained from personal files of enrolled women. All VHTs serving in the coverage area of five selected health centres were included in the group discussions. Group discussions included 5 VHTs each. Two VHTs each from the eight catchment health centres of the intervention arm were selected for key informant interviews. Ten professional health workers who were directly involved with the offer of prenatal, intra-partum and postnatal care in the intervention health centres were selected for key informant interviews. Different category of respondents and qualitative techniques were included to allow for data triangulation.

### Data collection

Five social scientists experienced in qualitative interviews and familiar with the local dialects, but not part of the study team conducted the interviews. Interview questions explored perceived benefits of the intervention from the perspective of VHTs, prenatal and postnatal women and professional health workers (see Additional files 1 and 2). Perceptions of postnatal women and VHTs towards recommended essential newborn care practices were explored (see Additional file 3). We explored women and VHTs opinions regarding adherence to non-application of substances on the cord, delayed bathing of the newborn for three days and initiation of breastfeeding within one hour and avoiding pre-lacteal feeds. Professional health workers were asked how they perceived the transfer of promotional maternal and newborn care responsibilities like home visits to offer health education to VHTs (see Additional file 4). Interview questions were open-ended to allow free expressions by respondents and further probing by interviewers. All interviews lasted 60–90 min.

A total of 67 interviews were conducted. Thirty six in-depth interviews were conducted with women; five group discussions with VHTs and 26 key informants (16 VHTs and 10 professional health workers). Four out of the initial 20 women could not be located during the postnatal period. Prenatal and postnatal in-depth interviews with the women were conducted in their homes. Group discussions and Key informant interviews were conducted at the health centre [See details in Table 1].

**Table 1:** Trends of data collection

s/n	Type of Interview	Participants	Target number	Number interviewed	Comments
1	First In-Depth Interview	Pregnant women	20	20	
2	Second In-Depth Interview	Just delivered women in no. 1	20	16	3 women had not yet delivered; 1 migrated
3	Key Informant Interview	VHT	16	16	
		Professional health workers	10	10	
4	Group Discussion	VHT	8 groups [4–5 VHTs per group]	5 groups	Saturation reached at five group discussions
	Total			67 interviews and 87 participants	

### Data analysis

Interviews were audiotaped and transcribed directly into English. Typed texts were read several times and exported to NVivo version 10. Analysis was by latent content analysis. As defined by H-F Hsieh and SE Shannon [22], latent content analysis involves subjective interpretation of text data through systematic classification process of codding and identifying themes or patterns [22]. This approach provides a deeper knowledge and understanding of the phenomenon under study in this case perceived benefits of the intervention. We preferred the *directed* latent content analysis because our analysis was based on pre-existing theories about home visitation by VHTs and mobile phone consultations, permitting the use pre-determined codes, also allowing for the emergence of new codes [23]. Predetermined codes included: perceived benefits of home visits and mobile phone consultations, perception and experiences of women and VHTs towards recommended maternal and newborn care practices; professional health worker perception regarding transfer of promotional maternal and newborn care to VHTs. Sub themes were developed under each code and summarized into meaningful text from which main themes were developed. Quotes were included to emphasis specific sub-themes (See Table 2 for summary of results).

**Table 2:** Analytical framework

		Codes	Sub Theme	Theme
Perceived Benefits of home visits and phone consultations	Women & Village Health Teams	• Perceived Benefits of home visits for the mother	• Mediated appointments between pregnant women/mother and health workers	• Promoted trust in the health system
				• Indirectly improved level of knowledge for VHTs
		• Perceived benefits of home visits for the newborn	• Facilitated access to care & additional information and improved the referral process	
		• General benefits of home visits	• Encouraged maternal and newborn service utilisation	
		• Perceived benefits of mobile phone consultation for the mother and newborn	• Phone consultations saved time and reduced transport costs	
			• Now we are famous in the	
			• community- Elevated social status for VHTs & Participant women	
				• Promoted confidence among VHTs, ingredient for sustainability of voluntary programme
		• Perceived benefit for the newborn	• Provided additional information and lead to attitudinal change	
			• Women felt better cared for than before the program began Elicited male partner support	
		• Perceived general benefits of mobile phone consultation		
Perception regarding recommended newborn care practices	Women & Village Health Teams	• Perceptions about newborn care-	• Nearly all VHTs practiced recommended practices Women & VHTs perceived as beneficial recommended maternal and newborn care practices	• Lived experiences are powerful tools for attitudinal change-prerequisite for behaviour change
		• none application of substance on the cord-		
		• delayed bathing for 3 days-		
		• initiation of breastfeeding within one-hour and avoiding pre-lacteal feeds		
			• Some VHTs doubted practicalities of delayed bathing and exclusive breastfeeding	
				• Recommended practices not accepted in minority of VHTs-may compromise the benefits of home visits
Perception regarding delegation of promotional maternal and newborn messages to VHTs	Professional health workers	• Beneficial	• Mostly accepted by professional health workers to delegate promotional maternal and newborn interventions	• Acceptable to work with VHTs
				• Sceptics about competencies of VHTs to offer maternal and newborn care
		• Not beneficial		
		• Maybe	• Caution about their mandate and how much they can perform regarding maternal and newborn care	
				• Fear of over “professionalising” VHTs
			• Abuse of the responsibility accorded to them	

In this analysis home visits by VHTs and mobile phone consultations were considered a single intervention. Therefore perceived benefits of the intervention were reported as effect of one intervention. Where applicable, views from different respondents considered to be describing the same sub-theme, whether convergent or divergent were presented under the same sub-theme.

### Ethical considerations

A written consent was secured at the time of enrolment of prenatal women into the community intervention study. The pre-enrolment consent detailed, home visits by VHTs and women’s participation in subsequent interviews. During this study women were reminded of their prior consent to participate in the study and verbal consent was obtained before conducting interviews. All women that were approached for the interviews accepted to participate. All VHTs and professional health workers provided verbal consent to participate in this study. Confidentiality was maintained throughout the interviews and results are reported to ensure anonymity. This research was approved by the Higher Degrees, Research and Ethics Committee of Makerere University School of Public Health, College of Health Sciences and the National Council of Science and Technology in Uganda.

## Results

Results are presented within three themes:

First, *Perceived benefits of home visits combined with mobile phone consultations.* Perceived benefits are presented within three thematic areas: i) improved access to maternal and newborn care, ii) additional information contributing to attitudinal change, iii), Perceived positive engagement of actors in maternal and newborn care (elevated social status for VHTs, women felt well cared for and enlisted male partner support.

Second*, Experiences and perception towards recommended newborn care practices* are presented along three sub-themes: *cord care*, *warmth, breastfeeding and avoiding pre-lacteal feeds*.

Third, *Perceptions of professional health workers towards transfer of promotional interventions to VHTs*. (see Analytical framework in Table 2).

### Perceived benefits of home visits and mobile phone consultation

#### Improved access to maternal and newborn care

Improved access to maternal and newborn care was considered to be an important prenatal benefit of the intervention. The intervention provided VHTs opportunity to mediate between prenatal women in the community and professional health workers at the health centre. Interaction between prenatal women, VHTs and professional health workers was perceived to encourage utilisation of facility delivery, provided accurate feedback from professional health workers and reduced the costs that prenatal women would otherwise incur in order to access care. VHT interaction with professional health workers experienced communication setbacks due to absence of professional health workers from their duty stations, failure to charge their telephone batteries regularly or system failures in telecommunication network.

One quarter of VHTs reported encouraging utilization of facility delivery in circumstances whereby women had decided to deliver at home:

*“The couple was planning to have a home delivery because they did not have enough money. I encouraged them to go to the health centre but they were not convinced, so I called the midwife then she talked to the woman and her spouse and they decided to go and delivered at the HC” (***KII VHT, Ikoba Health centre***)*

Mediation by VHTs between pregnant women and professional health workers was considered to be reassuring for the women and their families, especially when labour occurred at night or during public holidays. All VHTs reported having made at least one telephone contact with professional health workers to alert them of a referral.

*“When labour started, her father rushed to me. I called the midwife and she told the family to come to the health centre. She assured us that she was waiting for the woman. …on reaching there (*Health centre) *the midwife received and examined her (pregnant woman) and it was found that she was due (for delivery) …. That night she did not give birth … but gave birth the following day” (***KII VHT, Nyantonzi Health centre***)*

Some VHTs were of the opinion that phone calls have contributed to reduced incidences of maternal complications like miscarriages in their community because of the prompt attention that the women received. Sometimes health workers provided instructions to the VHTs on phone. Most VHTs perceived that the feedback that they received through phone calls improved the accuracy of information and were reassuring for the women and their families. For example,

*“…the phones have made communication and consultations so easy since the concerned person can be helped through the phone. Like Joy’s case (not real name), her baby had pus in the cord and the nurse advised me to clean it with salty water and the cord got healed” (***KII VHT, Kijunjubwa Health centre)**

Instructions given on phone from professional health workers to VHTs saved the women and their families of the long distance, the time and finances needed to reach the health centre. Nearly all VHTs (15/16) said that direct consultations through the use of mobile phones has helped them to reduce on the turnaround time needed to receive feedback since some of the consultations do not require physical movements of women to the health centre. Previously, VHTs were unable to offer immediate advice to the women in case of a difficult maternal or newborn condition. Now VHTs can get first-hand information from professional health workers without travelling to the health centre and this eased the VHTs’ work.

*“Phones have helped us because they saves time … It helped me because I was stuck when the woman asked me about excessive drinking of water during pregnancy I had to consult the nurse and she advised me what to say. It eased my work and so the woman continued to take water without any worries” (***VHT group discussion, Kijunjubwa Health centre***)*

It was not always the case that professional health workers could be reached on phone by VHTs or vice versa. On a few occasions professional health workers were found to be out of their health centres attending to workshops, or were away for official leave. In two intervention health centres the responsible midwives were away for maternity leave that lasted three months each. Instead the pregnant women were turned away and advised to seek for medical attention in private clinics. Conversely, professional health workers often failed to reach VHTs by phone. Failure to charge their phone batteries and loss of network were some of the common explanations given by VHTs. In either situation consultations for prenatal or postnatal women could not be completed successfully.

### Provided additional information leading to attitudinal change

Information about birth preparation obtained from VHTs during home visits was considered to be informative and led to attitudinal change among prenatal women. Nearly all the women (19/20) mentioned birth preparation as the most important maternal benefit for home visits made by VHTs. The same women confessed that previously they were not well informed about birth preparation especially about acquiring warm clothing for the newborn baby. About the topic that they liked most, three quarters of the women reported the topic about maternal nutrition which included eating a variety of food and birth preparation and saving some money for transport and emergency evacuation. A fifth of the women reported they understood better maternal and newborn benefits of using bed nets after VHT explanation. For example, they had acquired free mosquito nets during antenatal care visits but did not put them to use because mosquito nets caused excessive heat at night. They reported consistently using bed nets after learning about its benefits to their pregnancy and the newborn baby. Very few women (3/20) said now they seek medical attention from professional health workers unlike in the past when they would stay at home and use herbs or even go to retail drug shops operated by unqualified personnel.

*“Being my first pregnancy, I didn’t know that in case I fall sick I am not supposed to go to any drug shop to buy drugs …and avoid self-medication. After this teaching, whenever I felt sick I would go to the VHT and the VHT would call the health worker to inform her that I was coming to the health centre” (***IDI woman, Ikoba Health centre)**

Most health workers concurred with the VHTs that the women have shown a gradual change in attitude about birth preparation among prenatal women.

*“women now prepare well to receive their babies, they used to tell us that ‘’Ba eco bongo so mva alia siku’ literally means you cannot shop for unborn baby. You never know the baby may die… This idea is now going off people’s minds, they buy things like soap, clothes and other relevant items that can help them while here”* (**KII Health worker, Nyantonzi Health centre**)

### Perceived positive engagement of different actors in maternal and newborn care

Besides direct maternal and newborn benefits, the intervention was considered to contribute to emotional satisfaction among VHTs, prenatal and postnatal women.

### Elevated social status for VHT

In the groups’ discussions, VHTs expressed with enthusiasm how the intervention elevated their social status in the communities that they served. VHTs reported that being engaged in home visits and having direct telecommunication access to professional health workers led them to feel recognised as important stakeholders in the delivery of maternal and newborn care. VHTs felt that community members listened more to VHTs and they earned the respect of professional health workers.

*“Now we are famous because of our work. People listen to what I tell them, they respect us because we have brought services nearer to the people. If I go there (*to the health centre*) the health worker always welcomes me and she relates well with me, unlike in the past”***(VHT group discussion, Kigezi Health centre**)

In two group discussions VHTs reported that their calls to professional health workers were received with anger and this caused them to become reluctant to make future consultations with health workers.

### Women felt well cared for by VHTs and professional health workers

Prenatal and postnatal women who were visited in their homes considered that their health was given priority attention. About two thirds of the women conceded that it was the first time they were offered such detailed teaching on how to care for their pregnancy, about birth preparation and how to care for their newborn babies. These women reported that VHTs devoted time to explain maternal and newborn issues and VHTs allowed families to ask questions. All prenatal and postnatal women embraced the home visit programme. They considered the topics that were discussed by their VHTs to be relevant for their pregnancy and newborn babies.

*“It is a very good thing for VHT Primo (not real name) to visit me and my family to discuss health issues. I was so excited when the VHT visited me at home. I have never received such visit and information before, not even once. I want this program to continue even up to the next generation” (***IDI Woman, Nyantonzi health centre**)

However, home visit appointments were not honoured all the time by VHTs. One fifth of prenatal and postnatal women reported that sometimes VHTs did not keep their appointments for home visits causing anxiety among their family members. Only in one case, contrary to the protocol for home visits by VHTs, a woman revealed that her VHT invited the husband and her to go and receive health education from his home for which they obliged.

### Male partner support

Prenatal and postnatal women considered home visits as a useful means to persuade male spouses to make positive contributions towards their pregnancy, delivery and care for the newborn. This was because their spouses could receive first hand health education messages, unlike in the past where male spouses received second-hand messages through their female partners. One third of the women stated that their male partners got convinced about male-partner support for birth preparation and other psychosocial support needed during pregnancy, labour and newborn care. Prenatal and postnatal women further reported that home visits provided positive engagement of their male spouses in providing support during pregnancy, delivery and newborn care.

*“I want to tell you, before this teaching came (*referring to health education during home visits*), husbands knew that it was the woman’s role to save money and shop for the baby, but now husbands have once more assumed responsibility…” (***IDI Woman Nyantonzi health centre*)***

Previously, male partners did not take seriously the feedback from health centres, but home visits have transformed some of the men to cooperate and support their spouses during pregnancy. Male spouses seemed more likely to oblige to the VHT teaching compared to information received from the health centre.

*“When I told him (*spouse*) the teaching of the VHT he gave me money to buy all the requirements needed at the time of delivery. He also gives me transport to go for antenatal care sometimes he returns home with some of the food items that I feel like eating” (***IDI woman, Ikoba Health centre***)*

### Experiences and perceptions towards recommended newborn care practices

In this section we describe experiences and perceptions towards recommended newborn care practices (cord care, thermal care, initiation of breastfeeding and avoiding pre-lacteal feeds) from the perspective of postnatal women and VHTs. Postnatal women reported positive experiences with recommended newborn care practices. Lived experiences regarding recommended newborn care practices seemed most convincing for postnatal women and VHTs. However, few women and VHTs expressed mixed feelings about non-application of substance on the cord, delayed bathing for three days and avoidance of pre-lacteal feeds. Their perspectives are presented under the headings: cord care, thermal care and breastfeeding and the offer of pre-lacteal feeds.

#### Cord care

Nearly all women and VHTs accepted and perceived as beneficial for newborn health the recommended non-application of substances on the cord. Nearly all women interviewed accepted and practised non-application of substances on the umbilical stump. Women reported they relied on advice offered by their VHTs. The turning point for both women and VHTs was their lived experiences while caring for their newborn babies. Based on these positive experiences women and VHTs refuted earlier beliefs that application of substance on the cord facilitated healing as illustrated by this postnatal in-depth interview:

*“When the VHT taught me to use warm water only, I did not tamper with the umbilical stump or even the cord. I would only bath the baby and ensure that the cord is clean without applying any substances. In the past we were told to use herbs so that the cord can drop off quickly but when I cleaned it with warm water only, it also dropped quickly”* (**IDI woman, Ikoba Health centre**)

The demonstration from one VHT was even more illustrative because she delivered her baby during the time she was helping other women to care for their newborn babies, she said:

*“No we did not apply any substance on the cord, previously with other children, we used to apply herbs on the cord but for this one (*pointing to her baby*) we have stopped applying anything… This has given me the impression that the cord can heal even if no substance has been applied on it”* (**KII VHT, Ikoba Health centre**).

All VHTs interviewed said they were convinced that using clean instruments protected the newborn from contracting infections. Variously VHTs said, *“The cord is a very sensitive part of the baby’s body…”* “*dirty instrument may cause Tetanus…”, “used instrument may cause infections and even Aids Virus can be transmitted, that is if someone who used it was infected” “the baby can get other diseases and ends up dying”*. Only two of the sixteen VHTs were sceptical that the umbilical stump could heal without application of any substance. Sceptical VHTs suggested applying some baby powder, at least, to facilitate healing.

#### Thermal care

About three in four of women admitted to practising delayed bathing following the VHTs teachings. Some women said they tried to delay bathing of their babies for the recommended three days but succumbed to pressures from their spouses, highlighting the importance of spouses in the uptake of recommended newborn care practices.

Most VHTs supported the concept of delayed bathing and accepted that it keeps the baby warm and prevents them from catching pneumonia. Other VHTs explained that the baby’s body needs to get used to the environment around them before they can be bathed. One male VHT whose wife had a baby during this period admitted to practising delayed bathing for three days and their baby did not develop any problem implying that delayed bathing of the baby is possible without adverse consequences.

*“My wife delivered a month ago and the baby was not bathed for three days and the baby was fine, the baby did not smell. I think even if you do not bathe the baby nothing strange can happen”* (**KII VHT, Kigumba Health centre**)

Only three of the women considered that newborns bathing cannot be delayed for three days. This category of women perceived a newborns’ cry to mean discomfort due to heat and dirt. The women reported that the newborn stopped to cry once they have been bathed, evidence that it was dirt that was irritating the baby. Similarly, two out of sixteen VHTs considered as too long the suggested delayed bathing for three days. VHTs that were uncomfortable with the three days delays in bathing the newborn said they were willing to compromise and delay bathing only for one day:

*“Delaying to bath the baby is possible but three days is not possible may be only one day. How do you keep a baby for three days without bathing yet even me an adult I cannot stay for even six hours without bathing what about a baby taking three days?”* (**VHT group discussion, Kigumba Health centre**)

#### Breastfeeding and pre-lacteal feeds

Nearly all women accepted to initiate breastfeeding within one hour after delivery. The women said they learnt from VHTs that newborns were too young to take in anything other than breast milk. They were taught that “…*the first breast milk makes the baby brave*”. A few women reported they could not adhere to exclusive breastfeeding because other family members insisted on providing pre-lacteal feeds. Some women admitted they deliberately gave pre-lacteal feeds because there was no breast milk but stopped to give pre-lacteal feeds as soon as the breast milk became available. Similarly, one elderly VHT reported he would be compelled to recommend pre-lacteal feeds because the baby would be very hungry at that time when the mother’s breast milk is not yet coming. The rest of VHTs supported initiation of breastfeeding within one hour and were conversant with the benefits of exclusive breastfeeding.

### Perception of professional health workers

Nearly all health workers perceived as advantageous the transfer of promotional responsibilities to VHTs, because it allows professional health workers to concentrate on technical components of antenatal care such as palpation and screening for anaemia and HIV. Most professional health workers intimated that coordinating with VHTs has encouraged more women to make regular antenatal check-up because VHTs conducted surveillance for pregnant women and encouraged them to initiate antenatal check-up. The intervention did not require VHTs to conduct village surveillance for pregnant women rather wait for women who have been referred to them by professional health workers. VHTs took up the surveillance process out of their own initiative.

Two out of ten professional health workers considered the transfer of promotional responsibility to VHTs as unnecessary. These professional health workers perceived the entire responsibility of antenatal check-up and health education as part of nursing and midwifery and so delegation of such a role was not aligned to the policy of skilled attendance at birth. Some health workers cautioned that VHTs could abuse the delegated responsibility by claiming to be professionalised after the training and start to offer delivery care to women who are in labour. This could endanger the lives of women who are in labour.

*“I remember we trained Traditional Birth Attendants in delivery skills and they took up the delivery of pregnant women as a business from which they could make some gains, the mothers went to the TBA and some TBAs demanded for goats from the women and life goes by the way (*life is lost in the process*)* (**KII Health Worker, Kigumba Health centre**),

A few professional health workers took a compromise position to suggest that in the medium term when there is a shortage of health workers VHTs could remain relevant for the delivery of maternal and newborn messages in Uganda but were quick to suggest that once the staffing levels in the health system improved then VHT activities should be disbanded.

Six out of ten professional health workers reported that they were getting overwhelmed with paper work. Besides recruiting women into the intervention, they had other forms to fill such as Stop Malaria programme, return sheets for Infectious Disease Institute (IDI) and to make follow up with the VHTs.

## Discussion

This study of home visits by VHTs combined with mobile phone consultations with professional health workers for further advice in order to improve maternal and newborn care is, to our knowledge, new in sub-Saharan Africa. The study points to the potential of such an intervention for future community-based maternal and newborn care. Home visits and mobile phone consultations were generally perceived to positively influence access to maternal and newborn care and care-seeking through educational messages by VHTs, to prompt professional advice from health workers and to enhance referral in case of danger. Further, the intervention is considered to have contributed to motivation of VHTs and encouraged male partner participation in maternal and newborn care issues. However, the study highlights a number of bottlenecks such as the resistance among some VHTs to adhere to recommended newborn care practices, influences from family members to maintain unfavourable social norms such as provision of pre-lacteal feeds, absenteeism among health workers for a variety of (official and social) reasons, and irregular telephone connectivity attributed to limited availability of electricity for phone charging.

We came across the *philani* plus (+) studies conducted in South Africa which, like ours, deployed the combined use of mobile phones by community health workers with home visiting in order to improve maternal and infant health [24, 25]. However there were important differences between the *philani* study and ours. In our study the primary use of mobile phones was for consultations between VHTs and professional health workers. In the *philani* studies mobile phones were installed with a Geographic Positioning System (GPS) to locate respondents’ residences, and software with a checklist to ensure that community health workers exhausted their discussion topics. Mobile phones were used for data collection, data recording and transmission to a central server.

Home visit by VHTs and mobile phone consultations with professional health workers for advice is perceived to have a positive impact on maternal and newborn care and on care-seeking behaviour. This could have occurred though a series of pathways that resulted in trust and confidence among prenatal and postnatal women. Firstly, women received prompt information from VHTs, sometimes in consultation with a professional health worker. Moreover, the accuracy of these educational messages was improved by the use of distant mobile phone consultations with the health workers. Secondly, prior telephone appointments and referrals to the health facility ensured continuity of services from the community to the health centre facilitating the integration of preventive community and curative health facility services [26, 27]. Women also felt dignified by being visited in their home for matters regarding their pregnancy or their newborn baby. These elements may have led to improved trust and confidence in the health care system among women [28]. Furthermore, the perception by women of having received dignified attention and care contributes to more patient-centred-care (where patients are treated as unique individuals) which is an important element of quality of care [29]. Care that is more patient-centred is likely to improve utilisation of maternal and newborn health care services and adherence to recommended maternal and newborn care practices.

Our study indicates that VHTs are indeed capable of delivering accurate prenatal educational messages if backed up by professional health workers working in the public health system. Our findings are similar to studies conducted in India [30] and Bangladesh [31] where lay health workers provided maternal and newborn health talks to women and their families. These findings are encouraging for the promotion of VHTs as frontline structures in the Ugandan health system. The primary function of VHT is indeed to provide linkages (through community mobilization and advocacy) between the formal health system and communities. Mobile phone linkage between VHTs and professional health workers has the potential to contribute to this endeavour.

However, not all phone calls made by VHTs were attended to by professional health workers because they were either out-of-station, or their phones were not available on the network, or they were overloaded with extra work. When VHTs calls were not attended to, women and newborns could not receive the needed professional attention. The public service standing order for Uganda proposes deployment of one midwife at health centres of level III [32]. In case of official leave or illness of the midwife, antenatal consultations and delivery services may be suspended for the time that the midwife is out of the duty station. This obviously affects continuity of maternal and newborn services. Considerations should be made to ensure a minimum of two midwifes per health centre of level III or the deployment of multi-purpose health workers with midwifery skills to support the single midwife. The problem of limited electricity for regular battery charging is important in a rural setting like the one in our study. There are alternative measures to mitigate this problem. For example, the ‘solar suitcase’ has been used in places where electricity supply is irregular [33], but its cost (of about USD 1,500 per set) may be prohibitive. Cheaper alternatives such as the solar lantern and battery charger that cost about USD 10–20 may be feasible in the immediate and medium term.

VHTs reported an elevated social standing in their communities implying that their incentive to continue serving in a voluntary scheme could be sustained over a longer period. In addition, mobile phone consultations used by VHTs were perceived to increase VHT credibility, to indirectly improve their level of competence, and to enhance their appreciation by the communities they serve. This may contribute to boost the sustainability of a community program that hinges on voluntary inputs. Rendering community services with little or no compensation of time and effort, can indeed be an important challenge for the sustainability of Community Health Workers such as the VHT programme in Uganda. In this study, we did not register attrition among VHTs during the 14 months of our investigation. Aside from motivating factors like directly engaging with professional health workers through mobile phones, this maximum retention of VHTs during this intervention could be attributed to a number of factors. Firstly, ‘active’ VHTs were purposively selected because of their commitments in similar community programmes. Secondly, regular monthly meetings were held with the VHTs wherein most of the problems and complaints could be addressed. A modest transport refund of USD 5 was provided to them on a monthly basis. However, community-based programmes as this one should be mindful about the amount of task-shifting that is being made to VHTs, to avoid the risk of overloading work [34].

Home visits and phone consultations were considered to reduce the turnaround time to receive relevant maternal and newborn messages. Mobile phone consultation can save time, reduce the financial burden and other social costs that women would otherwise incur if they had to travel to the health centre to consult with professional health workers. This is encouraging because distance and cost of travels have often been cited as a limitation for utilization of maternal and newborn care services [35–39].

Acceptability and adherence to recommended newborn care practices among women and VHTs were high, suggesting that improved newborn care practices are taking root in the community. Acceptability and adherence to recommended practices is an important milestone towards behaviour change and a major ingredient for improved newborn care [40]. This finding further provides the evidence that home visits combined with mobile phone consultations has the potential to achieve desired newborn care practices. Studies conducted in south-east Asia have reported similar findings whereby women and family members adhered to preventive messages from lay health workers regarding maternal and newborn care [41]. These findings are encouraging because pre-intervention studies conducted in the same region two years earlier had shown poor newborn care practices [6]. Positive care experiences among women and VHTs is a powerful basis for behaviour change. Women that implemented the VHT teachings were confident that they would arrive at more favourable outcomes for their babies. Furthermore, VHTs’ practical and positive experiences with their own babies’ will contribute to dispel the myth that the umbilical stump can only heal when substances are applied on the cord.

However, our results also indicate that some VHTs stick very much to their traditional beliefs about newborn care, implying that the mere training of VHTs is not a guarantee for success. Although we report only 2/16 VHTs to be reluctant to adhere to avoiding pre-lacteal feeds and delayed bathing, one of these two VHTs also happens to be an influential locally elected leader in his community. VHTs will therefore benefit from regular support supervision visits and further education on the maternal and newborn benefits of adhering to recommended care practices. Exchanges with colleague VHT members that have had positive experiences of newborn care practices may also be helpful.

During this intervention, women reported improved male partner support during pregnancy, delivery and newborn care. The importance of male partner support in accessing maternal and newborn care has been widely documented [42–44]. Elsewhere, it has been demonstrated that little or non-involvement of male spouses leads to low utilization of reproductive health services [45]. However, the current antenatal care model in Uganda does not favour active male engagement in maternal and newborn care [46]. In many patriarchal societies male partners have better access to resources and they play a dominant role in resources allocation at the household level. A program that fosters male partner support is likely to reduce the incidence of gender-based violence, reduce the delay in decision-making to seek for care and to mobilize the resources needed to reach a health facility when a decision for skilled attendance has been made. In effect, male partner support can mitigate the first two delays of accessing care in the three-delay model. Broadly, male partner support will contribute to gender sensitivity at the family level.

Some professional health workers did have difficulty to accept that promotional responsibilities could be transferred to VHTs. These concerns reminds of the past controversy over training of TBAs for maternal and newborn care [47–50]. The challenge is to arrive at an optimum whereby the role of VHTs is to be considered as complementary, and not competitive, to the one of professional health workers. An institutional framework within the district local health system that will ensure synergistic implementation of promotional education at the community and technical care offered by professional health workers is to be considered.

### Methodological considerations

Different qualitative techniques were used and interviews were conducted among different stakeholders making triangulation of our data possible. Qualitative techniques provide insights that would otherwise not be obtained through quantitative techniques. Latent content analysis allows researchers to understand social realities in a subjective but scientific manner [51]. Initial coding is possible because of pre-existing research work and further probing on specific participants’ experiences is possible. As earlier stated, these findings will strengthen interpretation of quantitative results from the community intervention study.

Our results should be interpreted in the light of limitations of qualitative techniques: Interviews were conducted among a limited number of women in the intervention arm in the mid of the intervention; this may have introduced some selection bias in our results. On the other hand, the intervention was constantly assessed through regular field visits to ensure that the predesigned protocol was closely adhered to. Hence, time of selection of study participants may not significantly affect our results.

## Conclusions

At the point of the interim process evaluation, home visits and mobile consultation between VHTs and professional health care workers were well accepted and perceived to improve the implementation of newborn care practices. Results provide the indication that mothers and VHTs positively perceive the home visits and the mobile communication. The study shows that a mobile communication link between VHTs with health care workers can facilitate access to appropriate health care and reduce the delay in providing relevant maternal and newborn messages. Our study findings indicate that home visits by VHTs can create a more favourable environment conducive for appropriate and timely maternal and newborn care: it fosters male partner support, it improves VHT credibility in the communities that they serve and it makes women feel more dignified. Our study supports the hypothesis that home visits made by VHTs and backed-up by formal health care systems through the use of mobile phone consultation can contribute to prompt and accurate educational messages offered for maternal and newborn care. A number of challenges however remain to be addressed: there is the problem of prevailing social norms and practices, the logistic issue of phone charging, and the challenge of optimising the coordination between professional health workers and VHTs.

## Additional files

Additional file 1:
**In-Depth Interview Guide.**
Additional file 2:
**Second Interview: In-Depth Interview Guide-Women.**
Additional file 3:
**Question guide for VHTs.**
Additional file 4:
**Health Worker question guide.**

## Abbreviations

CHWs: Community Health Workers
FGDs: Focus Group Discussions
HIV: Human Immunodeficiency Virus
IDI: In-Depth Interviews
KII: Key Informant Interviews
MDG: Millennium Development Goals
TBA: Traditional Birth Attendants
USD: United States Dollar
VHTs: Village Health Teams
