# Supplementary material for: Use of mobile phone consultations during home visits by Community Health Workers for maternal and newborn care: community experiences from Masindi and Kiryandongo districts, Uganda
Source: BMC Public Health. 2015 Jun 18;15:560. doi: 10.1186/s12889-015-1939-3 (PMC4471930; doi:10.1186/s12889-015-1939-3)
Supplement: Additional file 1: — In-Depth Interview Guide. [file 12889_2015_1939_MOESM1_ESM.docx]

In-Depth Interview Guide

This interview will be conducted among women who have already delivered; have had an interaction with their VHTs during pregnancy and soon after birth

**Introduction**

This interview is about how you have been your experiences during the past pregnancies and your interaction with the VHT here in your village.

| Name of interviewee |  |
| --- | --- |
| Code Number |  |
| Age range |  |
| Number of children |  |
| Marital status |  |
| Parish |  |
| Village |  |
| Health centre of service |  |
| Interviewer |  |
| Date of interview |  |

Each mother will briefly introduce herself, where she lives, how many children she has etc

**Perceptions about the program:**

1. How did you join this programme?
2. How many antenatal visits did you make during the last pregnancy
3. How many times did the VHT make a visit to your home
4. What is your opinion about a VHT coming to visit you in your home to discuss issues of your pregnancy and your newborn baby with you and the rest of the family members? Is your husband supportive of this kind of arrangement?
5. Compared to receiving these health education talks from the health centre, which of the two would you say was better for you? Explain i.e. the VHT coming to your home and you going to the Health centre to get these talks?
6. Which discussion topics with the VHTs did you like most? Which discussion topics didn’t you like? Explain why
7. How confident are you after interacting with the VHT about your pregnancy and newborn baby? Explain

**Educational information offered by VHT and women’s attitudes**

1. Did the VHT talk to you about delayed bathing after giving birth (at least 2-3 days delay) what is your opinion about this suggestion? Did you accept or you refused the idea? Explain
2. Did the VHT discuss with you about not applying any substance on the cord? What was your reaction to this suggestion? Please explain
3. Did VHT discuss with you to prepare some of the necessary requirements for delivery and the newborn baby. Which things did she/he talk about? Did you find this a useful thing to do? Explain. Probe how it was helpful
4. Did the VHT discuss with you about initiating breastfeed after delivery? What information did he/she give you? (Initiate breastfeeding within one hour of delivery). What is your opinion about this recommendation

**Use of mobile phone**

1. Did you have any problem with your pregnancy? Or did you have any questions/doubts about your pregnancy for which you needed some clarification? What did you do about it? Did you see the VHT about it? What did the VHT do about it? Did he consult with the health worker at the health centre? How did he/she carry out the consultation?
2. Did the VHT have a mobile phone which she/he could use to consult the Health Worker? Did you benefit from this kind of arrangement where the VHT called the health worker for any problem or question that you had w?
3. Would you encourage VHTs coming to your home to offer information about pregnancy and newborn care? Explain

What about the issue of them having mobile phones where they could talk to HW at any given time?

1. What other suggestions would you make to improve the program of offering educational information to pregnant women and their families?

**Exploring previous pregnancies, delivery place and how decisions are made**

1. How many pregnancies have you had so far (include those that were aborted)
2. Please tell me where each of these deliveries took place
3. How did you arrive at this decision on where to have your delivery? Probe who were the people involved; and the influences
4. What were the reasons for your decision? Probe for past experiences or what they have heard
5. What was the outcome for the baby and mother (probe for any complications that might have arisen in the first month after delivery)
