# Supplementary material for: Use of mobile phone consultations during home visits by Community Health Workers for maternal and newborn care: community experiences from Masindi and Kiryandongo districts, Uganda
Source: BMC Public Health. 2015 Jun 18;15:560. doi: 10.1186/s12889-015-1939-3 (PMC4471930; doi:10.1186/s12889-015-1939-3)
Supplement: Additional file 2: — Second Interview: In-Depth Interview Guide-Women. [file 12889_2015_1939_MOESM2_ESM.docx]

**Second Interview: In-Depth Interview Guide-Women**

This interview will be conducted among women who were interviewed in November-December 2013 while they were still pregnant but now delivered. Respondent should have been visited by a VHT soon after delivery (within the first three days after delivery) [*please confirm this visit before you proceed with the interview*]

| Name of interviewee |  |
| --- | --- |
| Health centre of service |  |
| Interviewer |  |
| Date of interview |  |

Interviewer will remind respondent about the previous visit and indicate that this a follow-up visit. Seek for her consent to interview and audiotape the discussion.

1. **Delivery**

Please tell me what date and time that your delivery took place

Where was this delivery conducted from? What was the reason for making this decision about the place of delivery? [*Explore for the role of the husband, VHT or other family members in influencing this decision-making*]

1. **Care for the cord**

What material did you use for tying the cord? Who provided this material for tying the cord? Did the VHT influence the type of material used? How? Explain.

What material was used for cutting the cord? Who provided the instrument for cutting the cord? Did the VHT influence the type of instrument used? How? Explain.

Explain to me how you cared for the umbilical stump while at home. [Explore how it was cleaned, what substance was applied on the stump]. Did the VHT influence how you cared for the stump? How? Explain.

1. **Warmth**

How soon after delivery did you make the first bathing of the baby? [Please probe: *Here you want to explore how soon the baby was bathed - in hours/days the baby was first bathed*]. Did the VHT influence this decision? How? Explain.

1. **Breastfeeding**

What feeds did you give the baby soon after delivery, since there was no breast milk yet? [*You want to know whether pre-lacteal feeds were provided to the baby & why*]. Why? Explain.

How long after delivery did you start breastfeeding the baby? [*Please probe for precision of time since most of them may not have clocks*]. Did the VHT influence this decision? How? Explain.

1. **Observations**

**[Please observe & record your own voice while narrating what you have seen/observed]**

1. **Record what the weather** is like at the time of the interview (time of day, rainy, sunny, cold, warm etc). Wherever the baby is please request to see; observe for the following:
2. Was the baby sleeping or awake? Breastfeeding? , being bathed? Describe fully anything related to the baby at the time of the interview.
3. **Cloth covers** (blanket, head cap, socks, gloves, dress etc)
4. **Umbilical stump** (check whether there is any substance that was applied or not –do not reprimand if you find any material is applied-Ask what material it is and how is it meant to help the baby)
5. **Vaccination status**-ask for vaccination card-has the baby received Polio Zero (Polio 0) & BCG (check for the BCG scar in right upper outer part of arm)
6. **What is your opinion about practicing the following?**

***(Please probe for each response; pay attention to c-e where there is likely to be the highest resistance)***

1. Tying the cord with a clean cloth
2. Cutting the umbilicus with a clean instrument
3. Initiating breastfeeding within one hour
4. Not providing any pre-lacteal feeds to the baby
5. Not applying any substance on the cord
6. Delayed bathing for three days
7. **What was the greatest benefit you have had from the VHT since your pregnancy, delivery and baby to this time?**

**End**

**Thank you**
