# Supplementary material for: Use of mobile phone consultations during home visits by Community Health Workers for maternal and newborn care: community experiences from Masindi and Kiryandongo districts, Uganda
Source: BMC Public Health. 2015 Jun 18;15:560. doi: 10.1186/s12889-015-1939-3 (PMC4471930; doi:10.1186/s12889-015-1939-3)
Supplement: Additional file 3: — Question guide for VHTs. [file 12889_2015_1939_MOESM3_ESM.docx]

# Question guide for VHTs

**Introduction**

This interview is about the work you have been doing with pregnant women and their newborn babies here in the district of Kiryandongo/Masindi. You have been selected to participate in this discussion because of your role in the implementation of the program. You therefore have an experience of which we would like to learn from. In this discussions there are no right or wrong answers; I only want to hear your experiences and opinion about the program. The information that you provide will help us to review the program and even improve it.

| Name of interviewee |  |
| --- | --- |
| Age |  |
| Gender |  |
| Parish |  |
| Village |  |
| Health centre of service |  |
| Interviewer |  |
| Date of interview |  |

**Dialogue session with pregnant women and their families**

**Brief introduction of him/herself. This will help to fill the above table**

1. Please explain what this program that you are working on is all about [Normally VHTs have various programmes that they get involved in. Let’s give him/her a chance to mention those programmes and from her answer we see if ours is mentioned. After we bring the question to elaborate more about the programme of our interest]
2. Please describe how you have been going about your business of talking to pregnant women in this community (please provide specific examples) What do you do on this programme? Please give examples.
3. How did you first get in contact with the pregnant women in the community? How many pregnant women are you are currently following up in this program in your village?
4. Did you make contact with the rest of the family of all the pregnant women?[when you start close contact with them, who else do you talk to in their family? Please explain how you do it.
5. Please explain to me how you made contact with the rest of the family?
6. Can you describe how you were received by the pregnant/expectant women/mother?
7. How did the rest of the family members receive you? (Explore for reception by specific family members especially husbands, in-laws, auntie etc
8. If you did not make contact with some family members please explain why this was so
9. How did you arrive at the appointment date and time to make home visits to the pregnant women?
10. Did you consult all the relevant family members (like husband, mother-in-law, and auntie) before fixing appointments? Please explain why you chose the approach that you used.
11. Giving specific examples please tell me how many people you were meeting in the families during the dialogue sessions (please enumerate their relationships to the pregnant woman)
12. How would you describe the dialogue sessions? Were they all well conducted, or you think some were not well done? Please explain why you say so in each case. Provide specific examples to explain whether they went on well or did not go on well.
13. What are some of the topics that you discussed with the pregnant women and their families?
14. Overall, how would you describe the relevance of this program of providing information to pregnant women for the people of your community?[Do you think this programme Is helping the mothers in any way? Please explain.
15. How was the participation of the pregnant women and their families in this dialogue session? Did they tell you their own opinions? Did they ask questions? In each case please probe for specific examples
16. Were there family members who seemed to be more dominant during the discussions? Probe for which members;
17. Where there any issues that the family members were passionate about? Give examples.
18. How would you describe participation of the pregnant women during the dialogue sessions in the presence of other members of the family? [Were the pregnant women freely expressing themselves in the presence of other family members? Give examples
19. How did the other in-laws perceive the topics? (Probe: were they enthusiastic to participate or were they reluctant? Were they resistant?) probe for specific examples to support the answer
20. What about their husbands how did they perceive the topics that you were discussing with them? (Probe: were they enthusiastic, reluctant or resistant to participate probe for specific examples to support the answer
21. Would you encourage your wife, sister or other relatives to attend such sessions? Please explain.
22. Are you happy to participate in this exercise of talking to pregnant women in their homes? Please explain your answer [what motivates you to keep on going to speak to these mothers in their homes?
23. What have you liked about this programme? Are there aspects of this program that you have not liked? Can you now explain why you did not like them?[
24. From your own opinion how would you describe this program of talking to women about their pregnancy and newborn baby? Is it helpful to the pregnant women? Do you think we could copy this programme and take it to another district? Please explain your answer(s).
25. If you were to make any suggestions to improve this program of helping pregnant women what suggestions would you make? (Consider from the point of first being engaged as a VHT, coordination with the health workers, and coordination with the family members)
26. If you were to do it completely differently, how would you organize this idea of talking to pregnant women to help them understand better their pregnancy and caring for their newborn babies?

**Use of mobile phones**

1. How did you obtain this mobile phone?
2. When the mobile phone was given to you, how were you expected to use it? [Why did they give you this mobile phone?]
3. Please describe how you actually made use of this mobile phone?
4. How frequently have you used the mobile phone (probe either; in the last two weeks, in the last one month)?
5. Did you call the health worker in the last two weeks, or the last one month, or in the last two months, in the last three months?
6. Please provide specific examples of when and why you called the health worker using the mobile phone?
7. Did the health worker respond to your call immediately all of the time? Or did he/she call you later after missing your call? Or did she not respond sometimes
8. When you presented these problems to the health worker (please probe for each problem) please give me the different responses you got from the health worker
9. How would you describe the response(s) of the health worker towards your questions? (Probe-did he sound annoyed; happy to receive your call or showed little concern)?
10. Did you find all the responses useful for you and the women for whom you made the call?
11. Whenever you call the HWs, are they willing to listen to you and give you the necessary information that you need?
12. What are some of the things you did not like about the health worker?
13. How do you think the health worker can be made more useful in the use of mobile phones?
14. From your experiences of using this mobile phone so far, how would you describe the use of mobile phones to consult with health workers? Would you say it was useful all the time, sometimes only, or never useful at all? Please explain your answer
15. What are some of the things you have not liked with the use of mobile phones for consulting the health worker?
16. What suggestions would you make to improve the use of mobile phones to consult with health workers?
17. Would you recommend the use of mobile phones to be expanded to all VHTs in the district of Kiryandongo/Masindi? Please explain your response. What advantages do you foresee; or what disadvantages do you foresee?

**General perspectives**

1. Were there moments when you thought of quitting your participation in the programme? What had happened? How did you overcome and you chose to serve the mothers again
2. Are you paid to do this work in your village? What motivates you to continue to offer the services of talking to the pregnant women and their families? Please explain
3. How has this program enhanced your work as a VHT in this community? Probe-do you feel better empowered than before? Do you feel better recognized than before? Please explain
