# Supplementary material for: Use of mobile phone consultations during home visits by Community Health Workers for maternal and newborn care: community experiences from Masindi and Kiryandongo districts, Uganda
Source: BMC Public Health. 2015 Jun 18;15:560. doi: 10.1186/s12889-015-1939-3 (PMC4471930; doi:10.1186/s12889-015-1939-3)
Supplement: Additional file 4: — Health Worker question guide. [file 12889_2015_1939_MOESM4_ESM.docx]

# Health Worker question guide

**Introduction**

This interview is about the work you have been doing with pregnant women and their newborn babies here in the district of Kiryandongo/Masindi. You have been selected to participate in this discussion because of your role in the implementation of the program. You therefore have an experience of which we would like to learn from. In this discussions there are no right or wrong answers; I only want to hear your experiences and opinion about the program. The information that you provide will help us to review the program and even improve it.

| Name of interviewee |  |
| --- | --- |
| Age |  |
| Gender |  |
| Years of service post training |  |
| Parish |  |
| Village |  |
| Health centre of service |  |
| Interviewer |  |
| Date of interview |  |

**Continuity and permanence of maternal and newborn services**

**Please briefly introduce yourself and what you do at this health facility. For how long have you been working here?**

1. Please tell me, how many HW are in this facility and if all the gaps are filled as recommended by the MOH.
2. Are there any staffing gaps in this facility? Please explain.
3. How do you cope with this level of staffing/deployment assignment of duty

Explore further how the following services are offered in case the midwife is out of station for whatever reason

- 1. the offer of antenatal care (ANC);
  2. delivery of pregnant women and provision of educational information

1. How do you refer obstetric emergencies at your health centres? Probe for **transport**/evacuation – **vehicle costs**, **fuel** and who is **responsible** for all these logistical arrangements
2. How do you organize weekend and night duties here at the health centre especially for obstetric care (availability of staff to conduct delivery and organize emergency referrals)?

**Support to health services**

1. Do you get support supervision visits from the health sub district (HSD) or district?
2. When was the last time that they came to your health centre?
3. How long does the visiting team normally stay here at the health centre?
4. What exactly does the team do when they come for supervision at your health centre? Please probe each possible activities/intervention
5. What are some of the issues that you discuss with the supervision team when they come? (Probe whether they discuss about additional staffing especially midwives, accommodation for staff, electricity for lighting)
6. Have you had a particular visit regarding maternal health and or newborn health? When was it? Explain what happened during that visit? (please probe for details mentioned)

**The intervention**

1. Please explain what this intervention is all about?
2. How do you recruit the pregnant women into the study? Are there times when a mother resists taking part? What reasons do they normally give?

**Health worker interaction with VHTs**

1. Are VHTs involved in this particular programme? If so how?
2. Describe your relationship with the VHTs that are participating in this program of offering information to pregnant women and newborn care.
3. How do you link up with the VHTs in the community?

**Effect on access to information & care**

1. How do you think the VHTs visiting and holding health education discussions with pregnant women have contributed to the health and wellbeing of pregnant women? Do you have specific examples, please explain? (please encourage her tell the detailed story)Do you think the VHTs have contributed to the health and well being of pregnant women as they do their visits and health education? If so, please explain and tell me one scenario where you felt their impact on a mother
2. Have you been using mobile phones in this study? How have mobile phones been utilized in this program?
3. Please explain how you think the use of these mobile phones have contributed to the health of women and their newborn babies? (Probe for access to additional information; access to health care in case of emergency maternal or neonatal conditions)

**Problems introduced by the program**

1. How has this program affected your workload at the health centre? (probe for more administrative work; more women coming for ANC than before; more deliveries than before)
2. What are some of the challenges/problems that you have encountered in
   1. enrolling the pregnant women into this program
   2. connecting and communicating with the VHTs in the communities
   3. working with VHTs
   4. Using the mobile phones that you were provided with in this program?
3. For each of the four problem areas that you have mentioned please suggest any solutions to mitigate or solve the problem
   1. Enrolling of pregnant women
   2. Connecting and communicating with the VHTs
   3. Working with the VHTs
   4. Using the mobile phones that you were given for this program

**Perception towards program**

1. Would you recommend the use of VHTs to continue offering health education to the women and their families [in their homes] in the entire district Kiryandongo/Masindi? Please explain your answer
2. Would you recommend the VHTs to continue using mobile phones for the entire district of Kiryandongo/Masindi? Please explain your answer
3. If you were to reorganize this program how differently would you do it? Separately:
   1. VHTs offering educational information to pregnant women and their families
   2. VHTs using mobile phones to consult with the health workers
